# How does online postal self-sampling (OPSS) shape access to testing for sexually transmitted infections (STIs)? A qualitative study of service users

**DOI:** 10.1186/s12889-024-19741-x

**Published:** 2024-08-28

**Authors:** Tommer Spence, Alison Howarth, David Reid, Jessica Sheringham, Vanessa Apea, David Crundwell, Sara Day, Claire Dewsnap, Louise Jackson, Catherine H. Mercer, Hamish Mohammed, Jonathan D. C. Ross, Ann Sullivan, Andy Williams, Andrew Winter, Geoff Wong, Fiona Burns, Jo Gibbs

**Affiliations:** 1 Institute of Epidemiology and Health Care, University College London, London, UK; 2 Institute for Global Health, University College London, London, UK; 3 Barts Health NHS Trust, London, UK; 4 Lay representative, London, UK; 5 Chelsea and Westminster Hospital NHS Foundation Trust, London, UK; 6 Sheffield Teaching Hospitals NHS Foundation Trust, Sheffield, UK; 7 Institute of Applied Health Research, University of Birmingham, Birmingham, UK; 8 STIs and HIV Division, Blood Safety, Health Security Agency, Hepatitis, London, UK; 9 University Hospitals Birmingham NHS Foundation Trust, Birmingham, UK; 10 NHS Greater Glasgow and Clyde, Glasgow, UK; 11 Nuffield Department of Primary Care Health Sciences, University of Oxford, Oxford, UK

**Keywords:** Sexual health, Digital health, Testing, Qualitative, Access

## Abstract

**Background:**

Sexually transmitted infections (STIs) are a serious public health issue in many countries. Online postal self-sampling (OPSS) is increasingly used to test for STIs, a trend accelerated by the COVID-19 pandemic. There remains limited understanding of how service users experience OPSS and what leads them to access it over clinic-based services, or vice versa. This research seeks to address these gaps, by undertaking a large qualitative study which sits within the ASSIST study, a mixed-methods, realist evaluation of OPSS.

**Methods:**

Participants were recruited via clinic-based and online sexual health services in three case study areas in England. Purposive sampling was used to over-represent populations disproportionately affected by poor sexual health: young people; people of colour; men who have sex with men; and trans and non-binary people. Semi-structured interviews were analysed using Levesque’s conceptual framework of access to healthcare.

**Results:**

We interviewed 100 service users. Participants typically became aware of OPSS from sexual health services, the internet or word of mouth. Acceptability of OPSS was facilitated by the perceived privacy it offered over clinic-based services, which some participants found embarrassing to access. OPSS also enabled participants to overcome barriers to reaching clinic-based services, such as a lack of appointment availability, although difficulty obtaining OPSS kits in some areas undermined this. As all services in our case study areas were free to use, affordability did not significantly shape access, although OPSS enabled some participants to avoid costs associated with travelling to clinic-based services. Participants were usually able to engage with OPSS, finding it easy to use and reliable, although blood self-sampling was challenging for most. Participants valued the support offered by clinic-based services beyond STI testing, including the opportunity to access contraception or ask staff questions, and felt this was more appropriate when they had specific concerns about their sexual health, such as STI symptoms.

**Conclusions:**

Our findings constitute one of the largest qualitative studies to have explored OPSS and offer valuable insights to providers. OPSS shapes access to STI testing in a number of ways, including facilitating access in many circumstances, but users also want to retain access to clinic-based services, particularly for when they believe they need support beyond STI testing.

**Supplementary Information:**

The online version contains supplementary material available at 10.1186/s12889-024-19741-x.

## Background

Sexually transmitted infections (STIs) are a serious public health issue in many countries [1]. In England, diagnoses of chlamydia – the most common STI – are now stable, but syphilis and gonorrhoea diagnoses reached record levels in 2023 [2]. STIs in England are distributed inequitably across the population, with men who have sex with men (MSM), black ethnic minorities and young people aged 15–24 being disproportionately affected [2].

STI testing is crucial to enabling treatment and limiting onward infection [3]. Over the past decade, online postal self-sampling (OPSS) has emerged as an alternative to testing in sexual health clinics and other clinic-based settings. OPSS allows users to order a kit online, collect their own samples, post them to a laboratory for testing and receive results remotely [4]. Accelerated by the COVID-19 pandemic, when access to clinic-based services was restricted, usage of OPSS for chlamydia testing by young women aged 15–24 in England increased from 16% in 2018 to 43% in 2023 [2, 5]. This transition has occurred in the context of a wider effort to digitise healthcare, which has included a national recommendation that sexual health services in England provide OPSS [6]. OPSS services have also been introduced, and demonstrated strong uptake, in other high-income countries [7–9].

Despite this increase in usage, there remains limited understanding of what leads service users to access OPSS over clinic-based services, or vice versa. Uptake of OPSS has been found to be significantly higher among some population groups – such as heterosexual women, white people, MSM and those living in less deprived areas – than others [4]. Populations with lower uptake include black ethnic minorities and teenagers, both of whom experience high incidence of STIs. If populations which have lower uptake of OPSS also face barriers to accessing clinic-based services, then this could be leading to widening of health inequalities and increasing unmet need. Poor return rates for OPSS kits are also a cause for concern, with 52% of kits ordered from some services not being returned [10]. This is socially patterned, with heterosexual men and those living in deprived areas the least likely to use kits they have received [11]. There is wider evidence of certain populations being excluded by the shift towards digital healthcare, including some people with disabilities and those with fewer socioeconomic resources [12, 13].

**Fig. 1:**
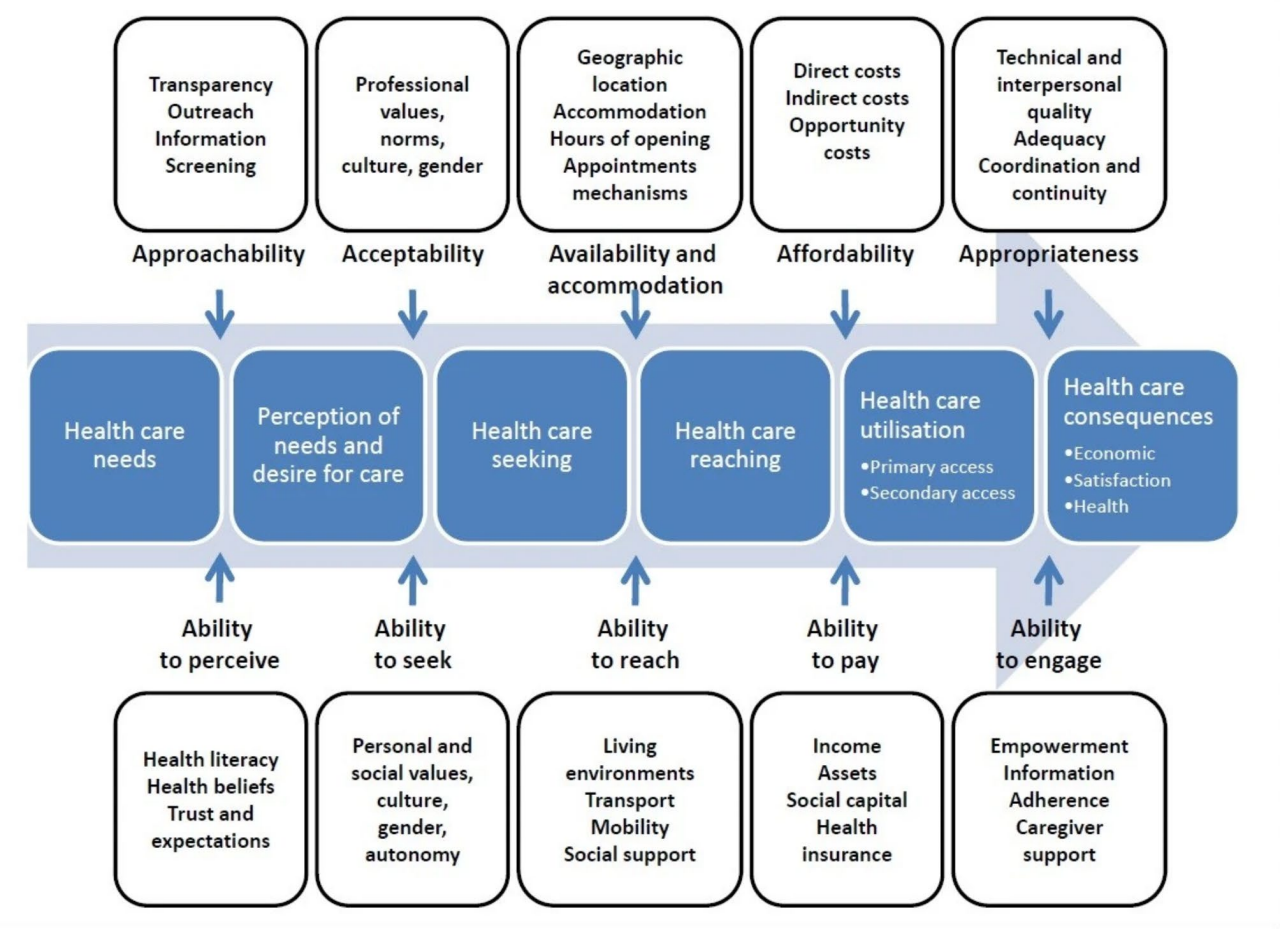
Conceptual framework of access to healthcare by Levesque et al. [14]

Access to healthcare is viewed by Levesque et al. [14] as *“the possibility to identify healthcare needs*,* to seek healthcare services*,* to reach the healthcare resources*,* to obtain or use health care services*,* and to actually be offered services appropriate to the needs for care”*. As set out in Fig. 1, they theorise that the ability of service users to progress through these stages of access is influenced by five dimensions of healthcare services: approachability; acceptability; availability; affordability; and appropriateness. Each of these dimensions corresponds with a parallel dimension of service user ability: ability to perceive; ability to seek; ability to reach; ability to pay; and ability to engage. This widely-used framework – which informed the design of our research – centres the perceptions and experiences of service users and allows facilitators and barriers to be explored, with a focus on socioeconomic determinants [15].

Existing research on access to STI testing within the context of OPSS is limited, with much of the literature exploring OPSS focusing exclusively on uptake [4]. Although several surveys have found high levels of acceptability of OPSS, these typically explore only the views of users who have successfully completed an OPSS pathway and are therefore affected by responder bias [7, 16–19]. They are also limited in how far they explore nuances in experiences. Qualitative research, which is well positioned to explore those nuances, has found that OPSS is acceptable to many people, in large part due to its perceived convenience and anonymity, but that many also have concerns around test accuracy, a lack of support when receiving results and inferior care compared to clinic-based testing [20]. Very few studies, however, have explored the experiences of users and those that have typically have small samples, or focus on a specific population or component of the OPSS pathway [20–22]. It is therefore challenging to know the applicability of these findings to other contexts and to understand divergent views.

This study seeks to address this gap by qualitatively exploring experiences of OPSS, alongside other sexual health services, and how this influences service users’ decisions on how they access STI testing. Unlike previous research, it explores OPSS in three case study areas, with large samples in each, allowing comparison of perceptions and experiences in different contexts. It also explores users’ previous experiences of sexual health services in considerable depth, giving insight into their routes to different services, and their experiences of the entire OPSS pathway. This includes access to care in clinic-based services, when participants were directed to these following the completion of STI testing.

## Methods

### Design

This research formed part of the ASSIST study, a mixed-methods, realist evaluation of the implementation and impact of OPSS [23]. One of the study’s objectives was to understand the impact of OPSS on access to care and the service user experience.

### Setting

ASSIST evaluated OPSS in three case study areas, labelled CSA1, CSA2 and CSA3 for anonymity. Although distinct in many ways, all three areas are urban and were selected in part because of their highly diverse populations, in terms of socioeconomic status, ethnicity, age and LGBTQ + identity. Each has a unique delivery model for sexual health services, including OPSS, which was launched at different times in each area. CSA1 operates OPSS, alongside a number of clinics, as part of an integrated sexual health service, which in England describes services set up to address most sexual health needs, including contraception, HIV pre-exposure prophylaxis (PrEP) and STI testing and treatment [6]. CSA2 operates OPSS as a standalone service, outsourced to a private sector partner. It is commissioned separately from, but works in partnership with, clinic-based integrated sexual health services across the city; two of these clinic-based services were selected as sites for this research, due to their high representation of populations of interest to this study. CSA3 delivers STI testing as part of a non-integrated sexual health service, with most contraceptive services in the area delivered separately and OPSS outsourced to a private sector partner. An overview of service provision in each area is provided in Table 1. OPSS and other STI testing is available free at the point of use to residents in all three areas.

**Table 1:** Overview of sexual health services in each CSA

	CSA1	CSA2	CSA3
**Overview of Services**	One sexual health service for area, offering OPSS and multiple clinic-based services	One OPSS service for area and multiple clinic-based services, delivered by a range of providers	One sexual health service for area, offering OPSS and one clinic-based service
**Clinic-based Service Provision**	Integrated	Integrated	Sexual health only; contraceptive services commissioned and delivered separately
**OPSS Delivery Model**	Delivered ‘in house’ by sexual health service	Outsourced to private provider – bespoke service	Outsourced to private provider – standard service
**OPSS Kit Availability**	Service users limited to one kit per month	Service users limited to one kit every three months	Limit of 20 kits per day on a first-come-first-served basis*
**OPSS Ordering**	Website, telephone or face-to-face in sexual health clinic or community setting (e.g. pharmacy, youth centre)	Website	Website
**OPSS Delivery**	Freepost or collection in sexual health clinic or community setting	Freepost or collection in sexual health clinic	Freepost
**OPSS Tests**	Chlamydia, gonorrhoea, syphilis and HIV; MSM also tested for hepatitis B	Chlamydia, gonorrhoea, syphilis and HIV; some users tested for hepatitis B and C, based on triage	Chlamydia, gonorrhoea, syphilis and HIV; some users tested for hepatitis B and C, based on triage
**OPSS Return**	Freepost or drop-off at sexual health clinic or community setting	Freepost	Freepost
**OPSS Results Delivery**	SMS	Online portal	Online portal

*This limit was introduced during our data collection; previously, service users were limited to one kit every three months

### Sampling and recruitment

We aimed to recruit 30–45 participants per case study area and used a purposive sampling strategy to ensure participant demographics over-represented populations which disproportionately experience poor sexual health. Our quotas for each case study area were: 3–5 MSM; 7–10 people from ethnic minority backgrounds; 14–20 people under the age of 25; and 3–5 trans or non-binary people. We sought to include equal numbers of men and women, irrespective of whether they identified as trans. We also purposively sampled people who had used either one or both of OPSS and clinic-based services, as well as those who had received an STI diagnosis from OPSS, in order to gain insight into this aspect of the user journey. All participants were required to be 16 years or over, to speak English and to have accessed online or clinic-based sexual health services within the past 12 months, in the three case study areas.

Participants were recruited via OPSS or in sexual health clinics between December 2021 and February 2023. OPSS users saw a link on the landing page of the service website or at the end of the OPSS kit request form, which invited them to express interest in the research. Clinic users saw recruitment posters or were approached by clinic staff. Potential participants were screened according to their age; gender; ethnicity; sexual orientation; and previous use of sexual health services. Potential participants who fulfilled quota requirements were contacted by TS or DR, who explained the study and arranged a time for interview if they were interested in participating. Participants completed an online consent form ahead of the interview and consent was confirmed again verbally at the start.

### Data collection

Data were collected via semi-structured interviews which explored participants’ use of the internet and online health services, their previous experiences of sexual health services (including, but not limited to, STI testing), their perceptions or experiences of the entire OPSS pathway (including ordering a kit, receiving and using it, returning it, receiving results and accessing treatment) and their perspectives on the appropriateness of different STI testing services in various contexts. The interview topic guide is provided as Supplementary Material 1. Participants had the choice of conducting their interview by phone, MS Teams or in person. They all received a £30 shopping voucher for participating. Interviews were conducted by TS, DR and AH, all of whom are professional researchers.

### Data analysis

Interviews were audio recorded and transcribed verbatim. The transcripts were reviewed for accuracy and to gain familiarity, before being pseudonymised, uploaded to NVivo software and coded using an inductive-deductive approach. This began with TS developing codes from the initial programme theory (IPT), developed by the research team as part of the wider realist evaluation, which hypothesised how service users would access and experience OPSS based on prior literature and clinician perspectives [23]. The IPT was developed from an initial logic model postulated by the research team before the study began (Supplementary Material 2), which set out to explain the introduction and impact of OPSS. It assumed that service users would perceive OPSS as: easy to find and access; convenient; easy to use and fitting in with 21st century life; providing privacy and minimising embarrassment or judgement by others; as ‘good’ a service as clinic-based services; and providing test results they could believe [23]. Additional codes were added inductively by TS from an initial sample of transcripts. Coding of the remaining transcripts was then undertaken by TS, DR and AH, who double-coded a selection of transcripts initially to check for consistency and codebook clarity. As analysis progressed, codes were continuously modified and added inductively. There were periodic meetings between TS, DR and AH to discuss new codes and their organisation into categories; meetings were also held with other members of the research team to discuss the analysis. The final code categories were organised into the dimensions of the Levesque et al. [14] conceptual framework of access, as it equipped us to understand the relationship between service users’ experiences of sexual health services and the determinants of using OPSS.

## Results

### Participants

We interviewed 100 participants. All participants chose to be interviewed by phone, aside from one who interviewed in person. Full demographic information is set out in Table 2.

**Table 2:** Participant demographics

Category	Number	
**Service usage**		
Clinic & OPSS	62	
Clinic only	14	
OPSS only	24	
**Case study area**		
CSA1	38	
CSA2	33	
CSA3	29	
**Gender**		
Cisgender Man	41	
Transgender Man	2	
Cisgender Woman	49	
Transgender Woman	1	
Non-binary	5	
Other	2	
**Age**		
Under 20	18	
20–24	26	
25–34	32	
35–44	14	
45–54	4	
55–64	5	
Over 65	1	
**Ethnicity**		
Asian	13	
Black	18	
Mixed	8	
White	57	
Other	4	
**Sexual orientation**		
Heterosexual or straight	57	(18 men, 39 women)
Gay or lesbian	19	(17 men, 1 woman, 1 identified in another way)
Bisexual	15	(4 men, 8 women, 3 non-binary)
Other	6	(3 men, 2 non-binary, 1 identified in another way)
Unknown	3	(1 man, 2 women)

### Analysis

Our findings are organised according to the Levesque et al. [14]. conceptual framework of access. The framework’s corresponding service and service user dimensions are presented together, giving five overarching determinants of access. For each, we have articulated a question that illustrates how we have applied the framework to OPSS.

*Approachability and ability to perceive: Could service users identify clinic-based and online sexual health services and what prompted them to recognise a need to access them?*

All participants were aware of clinic-based services, often viewing them as the default option for STI testing before becoming aware of OPSS. However, participants were also overwhelmingly aware of OPSS, in large part due to the efforts of services to promote it. They often discovered it while searching for STI testing services online, with OPSS often featuring prominently on search engine results, in social media adverts or on the websites of sexual health services:

*“I went onto the [sexual health service] website trying to get an appointment and they told me I could order a home kit instead. So*,* I just went with that option […] I think that’s where the first time I saw it [was].” (Participant 17*,* Black cis heterosexual woman*,* aged 25–34)*.

Staff in services raised awareness of OPSS, for example by directing participants to use it instead of attending a clinic, particularly when access to clinics was restricted during COVID-19 lockdowns:

*“I called the clinic up and thought I need to make an appointment*,* but the lady said oh*,* you can register for a self test online*,* and it will be out this week.” (Participant 62*,* White cis lesbian woman*,* aged 20–24)*.

Participants also reported being recommended OPSS as a test of cure, following treatment for an STI:

*“I went in to get tested*,* it did come back positive*,* so they said to test again to make sure it has gone before you see other sexual partners […] which you can do through an online testing kit*,* so that’s what I did.” (Participant 45*,* White cis gay man*,* aged under 20)*.

Alongside proactive efforts by services to increase awareness of OPSS, many participants reported learning about it via word of mouth, often from friends or new sexual partners:

*“I was talking to a best friend about it really because he’d done it before and he recommended how easier it was and stuff like that.” (Participant 53*,* White cis heterosexual man*,* aged 20–24)*.

The corresponding demand-side dimension of Levesque’s et al. [12] framework addresses service users’ ability to perceive services, which is shaped by factors such as health literacy and beliefs. Participants expressed a range of reasons that motivated them to access STI testing, many of which indicated a high level of sexual health literacy. New sexual partners were a trigger for many participants to get tested, usually because they had had a condomless sexual encounter – and were concerned about having contracted an STI – or because they wanted to transition to having regular sex without condoms. Some participants had tested due to a sexual partner notifying them that they had been diagnosed with an STI. Many also attempted to test regularly if they were sexually active, regardless of the status of their relationships:

*“I’ve had multiple friends in relationships that their partner has cheated on them and they’ve had chlamydia without knowing for a really long time. So*,* I’m a little bit of a hypochondriac where I’m like- I want kids eventually. […] So*,* if I get tested every three months*,* if I’ve had chlamydia for three months*,* it’s less likely to have a long term effect.” (Participant 28*,* White cis heterosexual woman*,* aged 20–24)*.

Participants were also aware that genitourinary symptoms were a reason to access testing and many had done so for this reason, either in their most recent testing experience or in an earlier one. However, they often believed that symptoms would mean it was more appropriate to get tested in sexual health clinics:

*“I think if I knew I had symptoms I would go to the clinic […] but I think if I just*,* if I’d just had a new partner*,* or just had unprotected sex or whatever*,* I’d probably get the self-testing kit. Particularly as you can still get the blood test for the HIV in the self-testing kit as well*,* so I feel like unless I was experiencing at the minute*,* symptoms*,* or there was […] another thing that was going on*,* I probably would get the self-test kit.” (Participant 48*,* White cis heterosexual woman*,* aged 25–34)*.

There were also participants who had less awareness of when or where to get tested, however, which was sometimes shaped by their cultural background. One participant, for example, only considered testing after her GP recommended that she do so:

*“I’m not really from the UK*,* I live in [country] and I’ve moved here recently and STI is not really a test people do unless you have to*,* like it’s not very common to do STI tests so I didn’t think about it.” (Participant 33*,* Asian cis heterosexual woman*,* aged 25–34)*.

*Acceptability and ability to seek: Did service users feel able to access clinic-based or online services within wider cultural and social norms?*

OPSS appealed to many due to the privacy they felt it offered over clinic-based services, and there were specific elements of the OPSS pathway which participants focused on as underpinning the privacy of the service, such as the discreet packaging kits were delivered in:

*“I thought it was very well packaged. So it comes in a brown box*,* it’s quite discreet. So I think if anyone was worried about*,* if they are living with friends or whatever their circumstances*,* as to oh someone is going to see that I am getting STD tested*,* it’s quite a discreet box.” (Participant 4*,* Asian cis gay man*,* aged 25–34)*.

Similarly, the opportunity to self-sample rather than have samples collected by a clinician appealed to many participants:

*“I’m quite a squeamish kind of person and I don’t like to be prodded and poked as everybody doesn’t. So*,* for me I was like oh yes*,* that sounds really good because you do it in privacy*,* do it myself and just send it off.” (Participant 73*,* Asian cis heterosexual woman*,* aged 25–34)*.

The option in some case study areas to collect OPSS kits, rather than having them posted, enhanced the perceived privacy of the service to some participants whose living situations meant that they felt they could not have a kit posted to their home:

*“My dad’s a bit nosy at times. So a box probably comes through the letterbox he’d probably most likely open it to see what it is. And then if he does do that*,* that will be difficult for me explaining to him what it is.” (Participant 76*,* Asian cis bisexual man*,* aged 35–44)*.

The perceived privacy of OPSS contrasted strongly with many participants’ perceptions of sexual health clinics, which they often felt required uncomfortable waits among other service users, or awkward interactions with staff. Concerns about stigma were raised by a number of participants, even some who had had positive, non-stigmatising interactions with staff in clinics:

*“I have got to say*,* the staff that I’ve come across at the NHS for sexual health specifically*,* have been absolutely wonderful. But I think with STI testing there is still societally such a stigma against it that I think when you are going for testing it’s invariable to have some of these […] anxieties.” (Participant 5*,* White cis heterosexual woman*,* aged under 20)*.

These concerns were also held about other clinic-based services, such as general practitioners (GPs). Although a number of participants had accessed STI testing opportunistically via their GP – and they did not feel the same concern about being seen in a GP practice, as no one in the waiting room would know their reason for being there – some still felt uncomfortable discussing sexual health with their GP:

*“There are times when I’ve been to the GP for stuff like that*,* you almost get the talk of like what you should be doing and what you shouldn’t be doing and that sort of thing. And whereas like they don’t do that at the sexual health clinics*,* which I think is*,* is what you’d want*,* like […] you are there for a reason*,* you don’t want to be like told off at the same time.” (Participant 48*,* White cis heterosexual woman*,* aged 25–34)*.

There were also concerns from some younger users that a GP they shared with their family may be less confidential, for example if test results were routinely sent to a parent’s mobile phone. There were participants, however, who felt a GP was the most familiar and confidential option, at least in circumstances when they felt they need to be examined by a clinician:

*“I go to the doctor’s for my contraception and […] smear tests and all of those things. So for some people if the clinic is somewhere where they already go to do all those other things*,* they might not have so much of a problem with going to the clinic whereas I don’t. I go to my doctor’s for those things. If I had symptoms*,* I would go to my doctor.” (Participant 52*,* White cis heterosexual woman*,* aged 45–54)*.

The communication of results was a component of STI testing which some participants felt compromised the acceptability of the service, particularly in the case study area which used SMS for this. Although participants were typically satisfied with results delivery, some had concerns that an SMS containing test results could be seen by others – a scenario which one participant had experienced:

*“I was sitting at dinner with friends and my phone was face up on the table. Everyone at the table had known that I was waiting on results so it wasn’t a big deal but had I been with others who weren’t*,* then they would have seen the results of my sexual health screening.” (Participant 10*,* Mixed ethnicity cis man*,* aged 20–24)*.

This concern was not expressed in relation to the two OPSS services which required users to log into an online portal in order to see their results.

The corresponding service user dimension of Levesque’s et al. [14] framework focuses on the ability to seek healthcare, within the context of societal norms and rights. As all of our participants had successfully accessed STI testing, this was not a strong feature of our data and there were contrasting views between participants about the role their identities played in access. There were many who felt their identities had no impact on which services they might use:

*“I’ve never felt like me being the skin colour I am or whatever is going to affect me wanting to go to a sex clinic or anything really. No*,* I’ve never felt that way.” (Participant 9*,* Black cis heterosexual man*,* aged 35–44)*.

There were a number of MSM, however, who felt that they were more easily able to access all STI testing services with less risk of stigmatisation due to cultural norms within their community and efforts by services to ensure they were inclusive:

*“Being Indian not really*,* I don’t think that has impacted anything. I think the one thing about being gay is that I think you are just more aware of the importance of sexual health and regular testing. I think it’s drilled into you quite early in your sexual experience*,* when you come out.” (Participant 4*,* Asian cis gay man*,* aged 25–34)*.

Trans and non-binary participants also often felt that sexual health services – whether OPSS or clinics – were more inclusive than more generic services offering STI testing, such as pharmacies:

*“Doing it through a pharmacy did mean that even though I was*,* my partner was a woman at the time*,* I was still being pushed contraceptives*,* which was not a pleasant experience […] I do think that if I was someone who was more sensitive to those issues it might have caused me distress. So, but like I have mentioned*,* I did really appreciate that [sexual health service] seems to be quite mindful about the gendered language that they are using.” (Participant 90*,* White bisexual non-binary*,* aged 25–34)*.

Although many appreciated that sexual health clinics were more trans-inclusive than other health services, trans and non-binary participants tended to prefer to test using OPSS, in part because it removed the risk of being misgendered or the burden of having to explain their gender identity.

Younger participants often felt they faced barriers accessing STI testing, although this led to different preferences in terms of service usage. There were some who were concerned about OPSS usage being identified, due to it arriving to their family home in the post, while others were more reluctant to use clinic-based services:

*“At that age*,* because it’s your first time approaching the topic of contraception […] it was a bit daunting. You go on your own*,* because you are a little bit shy*,* so it’s a bit daunting*,* going in person.” (Participant 85*,* Black cis woman*,* aged 25–34)*.

*Availability and ability to reach: How did the design*,* location and opening hours of sexual health services influence whether participants were able to access them?*

This was another clear and strong influence for our participants. Almost all perceived OPSS as relatively convenient, particularly in terms of time saved and reduction in travel compared to attending clinic-based testing services, such as sexual health clinics:

*“I am usually very*,* very limited for time*,* so […] I’ve missed maybe once or twice some appointments. So just the convenience of having a test kit come to your house*,* and you being able to test yourself*,* that’s […] very*,* very convenient. And then having to send it back*,* that’s pretty convenient.” (Participant 21*,* Black cis heterosexual man*,* aged 25–34)*.

Convenience also shaped how some participants chose to use OPSS, for example by posting their kit back for testing rather than dropping it off at a designated location:

*“[Posting is] the easiest way I think. I think anything else would require me to get in the car and drive somewhere or interact with someone or*,* you know*,* to a post office and have to queue up or whatever. Whereas that’s just straight there*,* dropped off*,* straight back.” (Participant 51*,* White cis heterosexual man*,* aged 25–34)*.

The perceived convenience of OPSS stands in stark contrast to most participants’ perceptions of sexual health clinics. There was a widely-held view, often based on personal experience, that getting an appointment at a sexual health clinic was extremely challenging or time-consuming:

*“I’ve not tried for a while but trying to get an appointment with [the sexual health clinic] was a little bit like trying to buy Glastonbury tickets. You have to be online at the exact right moment and you have to get lucky on top of that.” (Participant 26*,* White bisexual non-binary*,* aged 35–44)*.

Similarly, many participants spoke about the long waits which they expected or had experienced in clinics, even in circumstances where they had managed to get an appointment:

*“The wait was a lot longer than I thought it was going to be […] I had to wait for like three hours which was really kind of*,* inconvenient. And annoying.” (Participant 49*,* White non-binary*,* aged 20–24)*.

The time and costs associated with travelling to attend clinics were another inconvenience for some participants:

*“I was working and I’d have to take time out of work. I’d have to travel to the hospital. I’d have to have paid for parking. I’d have had to get there […] If I’d gone to a clinic*,* it would have easily took me maybe three*,* four hours to travel there and back*,* wait there.” (Participant 57*,* Mixed ethnicity cis gay man*,* aged 25–34)*.

There were participants who had experiences in clinics they felt were quick, however, for example in clinics which implemented effective processes to minimise waiting times:

*“After the first visit I seen [sic] one particular nurse and then he gave me a number*,* it might have even been a direct number to book into upstairs and the real good thing about that was there was no waiting times at all. So*,* if your appointment was at four you would get seen at four*,* so that I really*,* really liked.” (Participant 11*,* White cis gay man*,* aged 35–44)*.

There were also issues around the availability of OPSS in some circumstances, which presented a barrier to access. For example, one case study area saw delivery and processing times increase considerably during the COVID-19 pandemic:

*“I think the first time I got it*,* it did take a little while to get to my house. So*,* I was a bit annoyed*,* I wish I’d got it sooner in the post than I did […] I could have been seen to quicker if I’d just made an appointment.” (Participant 17*,* Black cis heterosexual woman*,* aged 25–34)*.

*“I’ve ordered the kit online and I think it took three months for the kit to arrive and it’s been three or four weeks since I’ve done the test and I’ve still not had the results.” (Participant 78*,* White cis heterosexual woman*,* aged 25–34)*.

Another case study area introduced a cap on daily OPSS orders during our data collection, which made it challenging for some participants to access this option:

*“The only issue I have is this time it said all the packs had been ordered but it came up like that for five times a day for three days on a roll*,* so I think they’d maxed out.” (Participant 54*,* White bisexual non-binary*,* aged 25–34)*.

Similarly, some OPSS services restricted how often users could order a kit. This was frustrating for some participants, who felt they were doing the right thing for their sexual – and for public – health but being impeded by the service provider:

*“The only bad thing about it is the fact that they don’t let you have more than a certain amount. Like the quota. And it makes you feel bad about yourself. It’s like*,* why won’t you just let me have a test? […] Because you’re trying to be responsible and get checked out.” (Participant 58*,* White cis heterosexual woman*,* aged 25–34)*.

*Affordability and ability to pay: What was the cost of accessing sexual health services and can users afford this?*

As all of the services included in this research were free to access, these dimensions were not strongly present in our data. However, many participants explicitly praised the fact that STI testing was available for free to them, particularly OPSS. This view was expressed particularly strongly by a number of people who were migrants to the UK when comparing sexual health services here to their countries of origin:

*“I was a bit shocked that they were free and they would pay for delivery then pay for the delivery back and also do all this stuff and it’s all like written out and it’s just a lot of effort. I was pleasantly surprised because we definitely don’t have those in [my country of origin].” (Participant 92*,* White cis heterosexual man*,* aged under 20)*.

There were also a number of participants who had used, or considered using, private OPSS services and valued that this was available to them for free:

*I did click on the [high street pharmacy] one first and I did look at all the different tests and the test for everything was like £120 […] If those tests had been cheaper and they’ve been like £15*,* I might have only got to that point and just gone*,* oh*,* well*,* for £15 I don’t have to go to a clinic. I don’t have to take time off work. I’ll just pay for that and just do it because that would feel like not that much money. (Participant 52*,* White cis heterosexual woman*,* aged 25–54)*

As noted earlier, some participants chose to access OPSS due in part to costs associated with attending clinic-based services, such as parking.

Although it did not necessarily shape their own access, some participants expressed a perception of limited NHS resources. This led a number to accept service standards which were below their desired levels:

*“Okay so I’d say that the walk-in clinics […] it’s a good and a bad. Like you’d wait for hours sometimes […] which obviously is not ideal. But at the end of the day I see it as*,* it’s a free service. And if there was more medical like staff to do it then yes*,* that would be the ideal world but we all know that we’re short staffed.” (Participant 14*,* White cis heterosexual woman*,* aged 20–24)*.

Some participants also expressed this view when discussing the wait for OPSS kits to be delivered in the case study area which experienced service disruption during the COVID-19 pandemic, although others were more critical of the delays.

There were also participants who chose OPSS over attending a clinic to preserve NHS resources for others they felt had greater need than them:

*“It frees up appointments for people that need them*,* because I know they only do a certain amount a day, which I completely respect*,* because you can only do so much in one day*,* and there’s people that will need in-person appointments a lot more than just me.” (Participant 80*,* White cis heterosexual woman*,* aged under 20)*.

*Appropriateness and ability to engage: Did services meet users’ health needs and did users have the capacity to do what services require of them?*

Participants typically felt that OPSS was appropriate to meet their needs, with many sharing that they felt it was able to provide results quickly and accurately:

*“I usually get those back within a week*,* week and a half*,* so it’s quicker to find out the results online which is [why] I use them […] I tested positive before so I know they’re obviously picking stuff up.” (Participant 55*,* White cis gay man*,* 25–34)*.

However, there was also a strong consensus among most participants that they should be seen in clinic-based in circumstances where they deemed themselves to be at higher likelihood of having an STI, such as when they were presenting with symptoms. This appealed to some participants as they felt it would enable them to be examined by a medical professional, to discuss their concerns with a clinician, receive quicker results and treatment, particularly in the case study area which experience delays in processing OPSS kits following the COVID-19 pandemic. Another appeal was the opportunity clinics offered to be tested for a wider range of STIs:

*“Postal testing is very limited […] if you go somewhere like [sexual health clinic] […] they search for a range of things. So trichomoniasis you can get tested for*,* BV because they have the laboratory*,* they test for thrush if it may be thrush.” (Participant 7*,* Black cis heterosexual woman*,* 25–34)*.

There were a number of participants who valued these qualities of clinic-based testing, irrespective of whether they were concerned they had an STI, although for many the convenience of OPSS superseded these positive aspects. There were also participants who felt that clinics enabled better access to further care, such as contraception, PrEP or vaccines for conditions like hepatitis B:

*“They talked about PrEP and were like*,* ‘Do you want to get on that? Go online and we can set up an appointment for you next week.’ So I’m actually going in next week to have my first PrEP appointment which is great. They were telling me about all the tests that they were doing and how I would get results back via text and that was great. I think the thing that surprised me the most was that I was able to get the vaccinations done right there and then.” (Participant 10*,* Mixed ethnicity cis man*,* aged 20–24)*.

Participants who had received a positive result via OPSS, however, often stated that they saw little difference in the pathway they subsequently followed. There were also some who had accessed additional care as a result of using OPSS:

*“I got the testing kit […] and it was great that it actually showed that I’m not hep B immune*,* which actually prompted me to get my vaccination this year.” (Participant 2*,* White cis gay man*,* aged 35–44)*.

There were also a small number of participants who shared that they had deliberately given false responses to order an OPSS kit, in order to avoid attending a clinic, even when they thought that it might be more appropriate for their health needs:

*“If you do have symptoms which ordinarily would require you to go to a [clinic] but […] there are no slots of*,* you know*,* convenient*,* in terms of time*,* you are more likely to say ‘Right well okay then*,* I will answer the questions in such a way that it does enable me to get this kit.’” (Participant 9*,* Mixed ethnicity cis gay man*,* aged 55–64)*.

The corresponding demand-side dimension of the framework addresses whether users have the ability to engage with a service. Most participants found OPSS easy to use, particularly in terms of ordering kits and – thanks to clear instructions – collecting urine, vaginal, oropharyngeal and rectal samples:

*“So for me [self-swabbing has] always been fine*,* really easy. Like no problems*,* like the instructions are there*,* it tells you what to do.” (Participant 58*,* White cis heterosexual woman*,* aged 25–34)*.

Participants were typically confident with their self-swabs and urine samples, although some said they would be more confident if they had been obtained by a clinician.

There was a widespread view, however, that blood self-sampling was prohibitively difficult and unpleasant. Lots of participants had difficulty obtaining enough blood, despite following the instructions in the kit closely. This meant some received invalid HIV and syphilis test results, leading them to attend a clinic for repeat testing:

*“It was very hard to get the blood out of. So I just ended up binning it because it was a nightmare […] It was painful […] I would have needed to literally slice my finger open and have the blood dripping in it for it to fill up.” (Participant 19*,* White cis gay man*,* aged 35–44)*.

There were a small number of participants who refused to use OPSS again following difficulties self-sampling blood, although others stated that it got easier with repeat usage and some had no difficulty at all. There were also a number of participants who accessed OPSS with a friend or partner, in some cases to get support with blood self-sampling:

*“One person I’ve helped do them quite a lot. We would find that her blood taking is really difficult. It takes ages. Whereas*,* for me it was really quick*,* my blood just came out straightaway. But for her it takes so long.” (Participant 35*,* Asian cis heterosexual woman*,* aged under 20)*.

Other participants spoke about previously attending sexual health clinics with friends, to overcome barriers such as embarrassment and anxiety, with one saying that OPSS enabled them to get tested when a companion was not available to attend a clinic:

*Interviewer: Okay. But previously*,* you’d always gone into the clinic. I guess*,* why did you choose to look for online services then?*

*Participant: Just because- I didn’t really go into the clinic all that often because I didn’t get tested previously. But […] I have anxiety*,* so I don’t really like going places on my own*,* and that. And obviously*,* I couldn’t always have someone with me. (Participant 60*,* Mixed ethnicity cis heterosexual woman*,* aged 20–24)*

## Discussion

We found that service users’ experiences of OPSS and other sexual health services shaped their future access to STI testing in a number of ways, as identified by the Levesque et al. [14] conceptual framework of access. Service users usually had a longstanding awareness of clinic-based services, while their awareness of OPSS typically came from being directed towards it by sexual health services, discovering it online or receiving an informal recommendation from a friend or partner. The acceptability of, and ability to seek, OPSS was facilitated by the perceived privacy it offers over clinic-based services, with many participants reporting that they felt embarrassed or uncomfortable when attending a sexual health clinic. However, MSM and trans participants often felt that specialist sexual health services – including OPSS – were inclusive towards them. The availability and ability to reach OPSS was predominantly influenced by its perceived convenience, as it enabled participants to avoid travel to clinics and waiting for an appointment, although difficulty obtaining OPSS kits in some areas undermined this. Affordability and ability to pay did not demonstrably shape access, as OPSS and clinic-based services were all free at the point of use, although some associated costs were a barrier to clinic-based testing, such as parking. In respect to appropriateness and ability to engage, participants generally found OPSS easy to use, aside from blood self-sampling, and were confident in its reliability, but felt that the holistic support offered by clinic-based services would be more appropriate in situations where they were particularly concerned about having an STI or another sexual or reproductive health issue.

Our findings are consistent with previous qualitative studies on OPSS, which have also identified convenience and privacy as strong facilitators to access [16, 18, 24–30]. As most previous studies had explored OPSS as a hypothetical scenario and did not include the perspectives of people who had experienced using OPSS, our findings add significant weight to the evidence indicating that these qualities facilitate access in practice. However, we also found that the ways in which OPSS is delivered can affect the impact of convenience and privacy as facilitators. Services which heavily restricted the availability of OPSS, for example, or which had long processing times for kits, were more likely to have users who felt clinic-based testing was a more convenient option.

Existing research has also identified concerns among some prospective users about self-sampling, such as worries about discomfort or samples being inaccurate. However, these were partially refuted by our data, highlighting the value of exploring the views of people who have used services [28, 31]. We found that participants overwhelmingly consider OPSS easy to use, including the vaginal, oropharyngeal and rectal swabs, and have confidence that these samples are sufficiently accurate. However, most participants struggled considerably with blood self-sampling, which offers insight to studies which have identified poor return rates for the blood component of OPSS kits [32]. Studies of prospective users have also found mixed views on the acceptability of blood self-sampling [27, 28, 30]. This research also identified facilitators to blood self-sampling, such as repeat usage, having access to multiple lancets and taking a sample with the support of another person. Our findings on participant satisfaction with self-swabbing and/or urine sampling highlight a limitation of our study: our sample’s relatively high health literacy. Research by Middleton et al. [21]. found that people with a mild intellectual disability saw the prospect of self-swabbing as overwhelming and challenging, something which we did not identify frequently in our data. However, to date there have been no studies exploring the experiences of people with mild intellectual disabilities who have accessed, or attempted to access, OPSS.

Our findings offer insight into how people enter and leave the OPSS pathway, a topic which has not previously been explored. As with previous research, exploring access to clinic-based sexual health services, we found that new sexual partners and symptoms were common prompts for participants to access testing [33]. However, we also found that sexual health services and social networks both played a significant role in enabling many of our participants to access OPSS, with some even doing so with friends or partners. This contrasts with some previous research which found that people often attempt to keep their use of sexual health services secret, even from friends, although some studies have also found social networks to be a route into accessing other sexual health services, such as PrEP [34, 35].

Access to further or more comprehensive care was part of the reason many participants valued the care on offer at clinics, at least in cases of high need, and there is some existing evidence supporting this perception. Bosó Pérez et al. [36]. found that people using remote sexual health services during the COVID-19 pandemic were less satisfied during more sensitive and emotional consultations, even though many recognised its value in other circumstances. Day et al. [37]. also reported challenges in identifying OPSS users who had experienced sexual assault, and providing them with adequate support. However, another study by the same research team found that OPSS operating procedures are effective at identifying and actioning safeguarding concerns with teenage users [38]. It is noteworthy that many of our participants who had tested positive for chlamydia or gonorrhoea using OPSS did not feel their treatment and care beyond this point was compromised or differed from what they would have experienced if they had tested at a sexual health clinic.

The communication of results is a key component of any STI testing pathway and there have been inconsistent findings from previous studies about service user preferences for how results are delivered. Research exploring the views of prospective users of OPSS had identified a range of preferred media for results communication, including SMS, email and phone, although with concerns among some about confidentiality and the lack of support from a healthcare professional [16, 24, 26, 30]. Studies which have explored users’ experiences of OPSS results have found high satisfaction with both online portals and SMS [22, 39]. Our findings corroborate this, with users of both methods typically expressing satisfaction. Although some users did have privacy concerns about SMS, it was not clear that this played a significant role in access.

### Strengths and limitations

This study is, to our knowledge, the largest qualitative exploration of OPSS, as well as the first to include users of different OPSS services. This allowed us to capture a wide range of perceptions and experiences, while also comparing and contrasting between different methods of delivering OPSS, in different contexts. The use of the Levesque et al. [14] conceptual framework of access enabled us to explore a wide range of facilitators and barriers to accessing both OPSS and clinic-based STI testing, including a number – such as participants’ awareness of, and ability to seek, OPSS alongside other testing options – which have received limited attention in prior research. The study sample was also highly diverse, as a result of our efforts to include populations of interest, such as young people, people of colour, MSM and trans people.

The study was limited by the fact that we found it difficult to recruit participants who had no experience of OPSS, despite this population being a key demographic in our sampling strategy. This was partially a consequence of the COVID-19 pandemic and its after-effects, which were ongoing during our data collection and had restricted access to clinic-based services. Staff in clinics also had limited capacity to support recruitment of service users who may not have accessed OPSS. We also found it challenging to recruit service users with low digital literacy, and did not analyse the impact of participants’ socioeconomic status, meaning we could not draw conclusions on these as barriers to access. As previously discussed, our sample typically had reasonably high sexual health literacy, as having tested for STIs was one of our inclusion criteria, and all participants were inherently comfortable discussing sexual health, having volunteered for the research. This made it challenging to explore these factors as barriers.

## Conclusions

Access to STI testing in the context of OPSS was shaped by a range of factors, including privacy, convenience, self-perceived risk, ability to self-sample and the opportunities users have to learn about OPSS. Commissioners and service providers seeking to improve access to STI testing should consider how the way services are delivered can reinforce facilitators to access, for example by minimising OPSS processing times and enabling kits to be collected as an alternative to home delivery, while also maintaining access to clinic-based testing for those who face barriers accessing OPSS. There would be value in further, targeted research exploring very marginalised populations whose perspectives have been insufficiently explored in the literature to date, such as those who are digitally excluded or who have never accessed OPSS, alongside populations which demonstrate persistently lower uptake of OPSS, including black ethnic groups, heterosexual men and people living in deprived areas.

### Electronic supplementary material

Below is the link to the electronic supplementary material.

Supplementary Material 1

Supplementary Material 2

## Data Availability

The data that support the findings of this study are not publicly available, as participants did not consent to this. Questions about the data can be directed to JG, Co-Chief Investigator, on jo.gibbs@ucl.ac.uk.

## Abbreviations

GP: General practitioner
IPT: Initial programme theory
MSM: Men who have sex with men
OPSS: Online postal self-sampling
STI(s): Sexually transmitted infection(s)
