# Supplementary material for: How does online postal self-sampling (OPSS) shape access to testing for sexually transmitted infections (STIs)? A qualitative study of service users
Source: BMC Public Health. 2024 Aug 28;24:2339. doi: 10.1186/s12889-024-19741-x (PMC11360737; doi:10.1186/s12889-024-19741-x)
Supplement: Supplementary file 1 — Supplementary Material 1 [file 12889_2024_19741_MOESM1_ESM.pdf]

Supplementary Material 1 Interview topic guide

ASSIST: Assessing the impact of online self-sampling for STIs & HIV

Service user interview topic guide

The interview will be conducted flexibly, using language familiar to the participant. Therefore, specific, instructions, questions, prompts and scenarios are not preset and are a guide.

***Preparation for interviewer**: familiarise yourself with the details of how OPSS offered in their service.*

Introduction to interview:

- Interviewer introduces themselves.
- Explain purposes of the interview, why the participant has been invited, and how the interview will run.
- Reassure participant about confidentiality of interview, and that we are interested in their views (there is no right or wrong answers)
- Explain that we anticipate the interview should take around 60 minutes
- Explain that we are interested in the following key topics:
    - Their experiences of accessing health information and services online (briefly)
    - Their experiences of accessing sexual health services
    - Their knowledge, experience and views on online sexual health delivery and postal self-sampling, considering the whole user journey (from ordering the test through to receiving results and further management)

Internet and smartphone use

- How do you normally access the internet?
  - Do you have easy access to WiFi or mobile data?
- What do you do online that's related to health?
  - Do you ever look up information about health online? Where? When? Why?
  - What health services have you accessed online?
    - Have you been tested for Covid-19 using a kit that you ordered online? If so, can you tell me about that experience?

Previous experiences of accessing sexual health services

- I understand that you've been tested using [OPSS/a clinic/both] – is that correct?
  - Did you order an online self-sampling kit on this occasion?
- When was the first time you used sexual health services?
  - Why did you choose to get tested on that occasion?
- What other sexual health services have you used?
- How did you find using them?
  - What did you feel worked well about them?
  - Was anything better than you expected?
  - What could have been improved?
- How often to you usually use [different] sexual health services?
- What normally prompts you to get tested for STIs?
  - Are there any other circumstances you think would lead you to get tested for STIs? (For example, specific sexual encounters or symptoms.)
  - Would you choose to test in a different way depending on why you were getting tested?

Experience of accessing online postal self-sampling services [only if known experience from screening questionnaire]

- Talk through their experiences of accessing online postal self-sampling, covering each component of the user journey.
    - Exploring expectations, emotional responses, what happened etc

Accessing

- When was the first time you used online self-sampling?
- How did you first hear about it?
- What made you decide to use it?
    - [We want to go beyond rationale and understand] How were you feeling when you decided to order a kit?
    - What made you decide to use an online self-sampling kit instead of going to a sexual health clinic?
- Would you have chosen to get tested in a different way if you could? Why?

Ordering

- How did you order it?
- How did you find the order process?
    - Is there anything you would change about it?
    - What did you think of the questions you were asked?
- [If relevant] How did you choose to receive your kit? Why?
- How did you feel when you received the kit?

Sampling

- Tell me about using your kit to take your samples.
    - When did you open the kit? Why did you choose to do it then?
    - How did you feel when you opened the kit?
- Was the kit different to what you expected? How?
- How did you find taking your:
    - Urine / Vaginal Swab?
    - Finger-prick blood sample?
    - [If relevant] Rectal and/or throat swab?
- How confident were you in your samples?

Returning

- [If relevant] How did you choose to return your kit? Why?
- How did you feel when you posted your kit?
- Would you want to return your kit in a different way, if you could?

Results

- When did you receive your results?
- How were your results delivered to you?
  - Why did you choose this and not the other options?
- How did you find the wait?
  - How did it affect your decisions about your [sex life/sexual behaviour]?Did if affect whether you had sex?
  - Did it change how you felt about having sex?
  - Did it affect who you considered having sex with?
- How did you feel when you received your results?
- Did you feel confident in your results?
- Is there anything you would change about how results are delivered?

Post-testing

- What happened after you received your results?
- What were your results?
- Did you receive any treatment? If so, how? How did you find that experience?
- Did you speak to anyone at the sexual health service? What about? How did you find this?
- Did you use any other sexual health services?
  - Contraception
  - Partner notification
  - PrEP
  - Vaccination
- How did you feel about the information you received [with/after your] results?
- What support do you feel you needed after receiving your results?

- What could have been improved about your experience following receiving your results?
- How did you find your previous experiences receiving results from STI testing?
  - How did they compare to the way you received results on this occasion?
  - What worked well about them? What could have been improved?
  - Have you ever received a [positive/negative] result remotely? How did you find this? How does receiving results remotely from OPSS compare to receiving results remotely after being tested at a clinic?
- How did it affect your decisions about your [sex life/sexual behaviour]?
  - Did if affect whether you had sex?
  - Did it change how you felt about having sex?
  - Did it affect who you considered having sex with?

If they have accessed OPSS since their first experience of it:

- How was your subsequent experiences of online self-sampling been different to your first experience? Have you had any experiences of online self-sampling which have been different to the others?
- Have you changed how you get tested for STIs since your first experience of online self-sampling?
- How do you feel about online self-sampling now compared to how you felt when you first used it?

If they have previous experience of being tested in face-to-face settings:

- How does using online self-sampling compare to your experiences of being tested in a clinic [or other face-to-face setting]?
  - What do you think is better about getting tested in a clinic?
  - What do you think is better about using an online self-sampling kit?
- Why did you decide to use online self-sampling instead of being tested in a [clinic/face-to-face setting] again?

If unaddressed by earlier answers:
- Tell me what you thought about privacy when you accessed online self-sampling.   - How would you compare the privacy in clinics compared to online self-sampling?   - Would you change anything to do with privacy and online self-sampling?   - Tell me what you thought about the convenience of online self-sampling.   - Are there any aspects of online self-sampling you find particularly convenient?   - Are there any aspects of online self-sampling you find inconvenient?   - How would you compare the convenience of getting tested in a clinic to getting tested via online self-sampling?   - How does the convenience of online self-sampling for STIs compare to other online health services? What about online services generally?   - We're particularly interested in finding out how ethnicity, gender and sexual identity [and migrant status] might affect how people use sexual health services. You've told us you identify as…   - How do you feel being [gender] might have affected your experience using different sexual health services?   - How do you feel being [ethnicity] might have affected your experience using different sexual health services?   - How do you feel being [sexuality] might have affected your experience of using different sexual health services?   - How do you feel your experience as a migrant might have affected your experience using different sexual health services?   - Do you think any other aspects of your identity have made you more or less likely to prefer online self-sampling?   - Have you ever felt that your identity has affected how you were treated in a healthcare setting?

Concluding questions

- Is there any additional support you would have liked while using online self-sampling? How would you have wanted to access this (e.g. phone/web chat)?
    - Was there any support you did access which you found useful?
- What advice would you give someone who lives in your area and is thinking about getting tested for STIs?
    - Have you ever recommended OPSS or another sexual health service to someone?
    - Has anyone ever recommended a sexual health service to you?
- What advice would you give someone who is thinking of using an OPSS kit for the first time?
- What advice would they give to someone wanting to design or improve an online postal self-sampling service in their area?
- Is there anything else you would like to raise?

Knowledge and views of online postal self-sampling services [if no known experience from screening questionnaire]

- Are you aware of being able to access online self-sampling in your area? If yes:
    - When/how did you first hear about it?
    - What are your thoughts on it?
- Is this something that you would consider using? Explore reasons for responses.
    - How do you think online self-sampling would compare to being tested in a clinic?
- Talk through how online postal self-sampling works in their area, covering (for example) the following components which are shared verbally and/-visually [depending on whether phone/video/face-to-face interview]:
    -

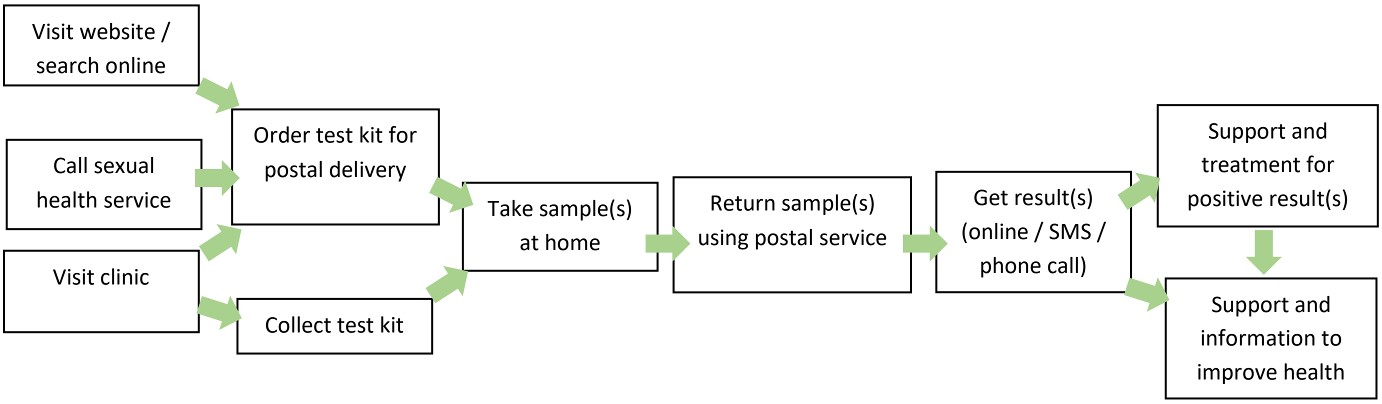

- Explore what they think about each part of the user journey:
    - How would you feel about ordering a kit? Which way might you do it and why?
    - What do you think the process would be of ordering a kit?
    - What do you think about receiving the kit by post?
        - What do you think about collecting the kit at a clinic?
    - What do you expect the kit would be like?
    - What samples do you think you would have to take?
        - How would you feel taking these?
    - What do you think about receiving results by SMS/online/phone? Which might you choose and why? Why not the others?

- Are there any parts of this pathway that you have actually experienced (e.g. getting results via text)? How did you find this?
- Which aspects of the pathway would you find most appealing? Why?
- Which aspects of the pathway would you find most off-putting? Why?
- What support would you like were to use this pathway? How would you want to be able to access this (e.g. phone/web chat)?
- What advice would you give to someone wanting to design or improve an online postal self-sampling service in your area?
- We're particularly interested in finding out how ethnicity, gender and sexuality [and migrant status] might affect how people use sexual health services, as other studies suggest it can be harder for some groups defined in these ways to access care. You've told us you identify as…
    - How do you feel your gender might have affected your experience using different sexual health services?
    - How do you feel your ethnicity might have affected your experience using different sexual health services?
    - How do you feel your sexuality might have affected your experience of using different sexual health services?
    - Do you think has any aspects of your identity have made you more or less likely to prefer online self-sampling?
    - Have you ever felt that your identity has affected how you were treated in a healthcare setting?
- Anything else you would like to raise?

Scenarios [for all whether experience or no experience of OPSS, if helpful to explore participant's thoughts further]: interviewer to introduce concept of scenarios for using OPSS vs clinic-based services. Interviewee will be invited to talk through thought process for different scenarios, focusing on how the person might choose to get tested, why and what their thoughts and feelings would be about the process. Examples used will be guided by the previous experiences of the participant, and will include:

1. Someone who has had a 'one night stand' with a new sexual partner and wants to get tested for STIs
2. Someone who has been notified that someone they have had sex with has an STI.
3. Someone who doesn't have symptoms and gets tested regularly.
4. Someone who will find out they have tested positive for:
    a. chlamydia
    b. HIV.
5. Someone accessing services for the first time.
6. Someone who has phoned the clinic with vaginal discharge (women) or mild irritation/pain passing urine (men) and been told that they need to test online.

Ending:

- Thank participant for their time.
- Check if any questions.
- Check if ok to contact them on the number or email address provided if there are any points that require clarifying.
- Voucher
- Provide with information about support services that they can access.
