# Supplementary figures and images for: How does online postal self-sampling (OPSS) shape access to testing for sexually transmitted infections (STIs)? A qualitative study of service users
Source: BMC Public Health. 2024 Aug 28;24:2339. doi: 10.1186/s12889-024-19741-x (PMC11360737; doi:10.1186/s12889-024-19741-x)

## Supplementary Material 2 Logic model explaining the introduction and impact of OPSS services (23)

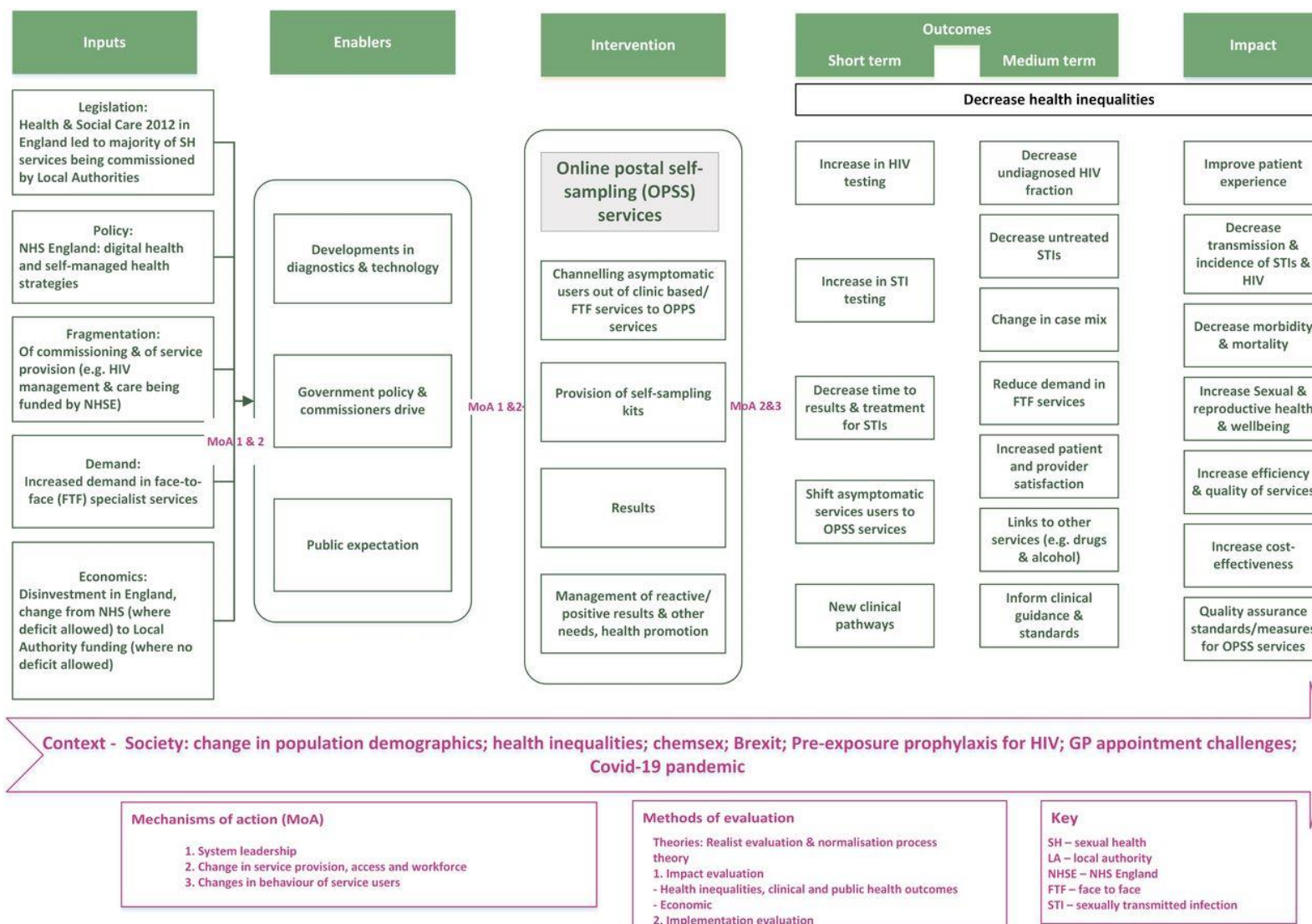

Supplement: Supplementary file 2 — Supplementary Material 2 [file 12889_2024_19741_MOESM2_ESM.pdf]
